# Trajectory and Demographic Correlates of Antibodies to SARS-CoV-2 Nucleocapsid in Recently Infected Blood Donors, United States

**DOI:** 10.3201/eid2907.230173

**Published:** 2023-07

**Authors:** James M. Haynes, Roger Y. Dodd, Lauren A. Crowder, Edward P. Notari, Susan L. Stramer

**Affiliations:** Author affiliations: American Red Cross, Rockville, Maryland, USA

**Keywords:** COVID-19, SARS-CoV-2, severe acute respiratory syndrome coronavirus 2, coronaviruses, viruses, nucleocapsid, spike protein, antibody, N antibody, S antibody, waning, half-life, blood donors, coronavirus disease, respiratory infections, trajectory, demographic correlates, zoonoses, United States

## Abstract

We evaluated antibodies to the nucleocapsid protein of SARS-CoV-2 in a large cohort of blood donors in the United States who were recently infected with the virus. Antibodies to the nucleocapsid protein of SARS-CoV-2 indicate previous infection but are subject to waning, potentially affecting epidemiologic studies. We longitudinally evaluated a cohort of 19,323 blood donors who had evidence of recent infection by using a widely available serologic test to determine the dynamics of such waning. We analyzed overall signal-to-cutoff values for 48,330 donations (average 2.5 donations/person) that had an average observation period of 102 days. The observed peak signal-to-cutoff value varied widely, but the waning rate was consistent across the range, with a half-life of 122 days. Within the cohort, only 0.75% of persons became seronegative. Factors predictive of higher peak values and longer time to seroreversion included increasing age, male sex, higher body mass index, and non-Caucasian race.

Blood donors are a healthy subset of the population, and data collected from testing their donations for infectious disease markers provides opportunities for monitoring epidemiologic characteristics translatable to the general population (*1*,*2*). Most blood donors return to donate frequently and at somewhat regular intervals, which offers a unique opportunity for longitudinal studies. In June 2020, the American Red Cross and many other large blood centers began testing donors in the United States for antibodies against SARS-CoV-2 as a means to identify sources for convalescent-phase plasma and to attract potential donors (*3*).

Natural decay of SARS-CoV-2 antibodies is expected after vaccination and natural infection and can be quantified by serosurveillance over time. Specifically, antibodies to the spike protein develop after vaccination or infection and can take a relatively long time to decrease, but antibodies to the nucleocapsid protein develop only after natural infection and wane at a faster rate, which is particularly detectable with testing by using an IgG assay format versus a total antibody assay format (*3**–**8*).

In this study, over ≈1 year, every blood donation was tested for antibodies to SARS-CoV-2 spike protein antigens. Donations that had a reactive result were further tested for antibodies to nucleocapsid antigens. A subset of donors who seroconverted during the study period and who donated at least once more were investigated. Signal levels and waning rates for nucleocapsid antibodies were modeled, and demographic correlates, including age, sex, body mass index (BMI), and race, were sought.

## Methods

During June 2020‒June 2021, the American Red Cross tested >5.2 million blood donations from 2.4 million blood donors for the presence of total antibodies to the SARS-CoV-2 S1 protein by using the Ortho-Clinical Diagnostics VITROS Anti-SARS-CoV-2 Total Reagent Pack test system (Thermo Fisher Scientific, https://www.thermofisher.com). Reactive samples were tested for total nucleocapsid antibodies by using the Roche Elecsys Anti-SARS-CoV-2 test (Roche, https://www.roche.com), as described elsewhere (*3*,*9*). By manufacturer guidelines, a signal-to-cutoff (S/CO) value >1 was considered reactive. A total of 1,527,776 donations were spike protein reactive, of which 475,943 (31%) were subsequently classified as nucleocapsid reactive.

The cohort selected for the study was nonreactive donors who seroconverted to reactive for antibodies to spike protein and to nucleocapsid at the same time, or within no more than 120 days, and who had at >1 donation after seroconversion (Table 1) (*3*). We identified persons only by code and were advised of reactive test results; individual characteristics were self-reported and obtained from routine blood donation records. This study was approved by the American Red Cross Biomedical Services Institutional Review Board.

**Table 1:** Cohort selection process for study of trajectory and demographic correlates of antibodies to SARS-CoV-2 nucleocapsid in recently infected blood donors, United States, June 2020‒June 2021*

Characteristic	No. donors (no. donations)
Donations received during June 2020‒June 2021	2,448,127 (5,221,553)
S1 reactive tested for N antibody	1,092,736 (1,527,776)
N reactive	321,387 (475,943)
Seroconverters, N antibody reactive after N antibody nonreactive	54,414 (190,983)
Period to observed N antibody peak value <120 d from N antibody nonreactive	32,376 (126,227)
Donations with N antibody signal value reported	32,376 (89,682)
Observations at and after observed peak	32,376 (61,432)
Donors with >1 observation after observed peak	19,326 (48,382)
Observations that were valid or without apparent reinfection	19,323 (48,330)

*N, nucleocapsid; S, spike.

### Peak Signal/Cutoff Measurement and Antibody Waning

**Figure 1:**
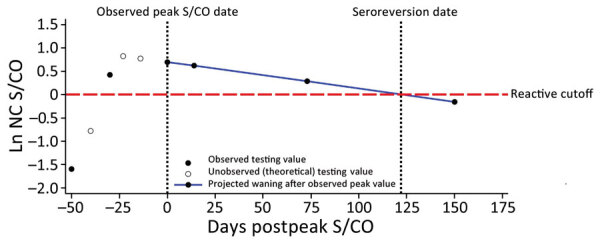
Potential nucleocapsid antibody test signal over time, showing likely sequence of S/CO values and seroconversion and seroreversion for nucleocapsid antibodies, in study of trajectory and demographic correlates of antibodies to SARS-CoV-2 nucleocapsid in recently infected blood donors, United States, June 2020‒June 2021. Each circle shows a potential value; solid circles indicate observed testing values and open circles theoretical unobserved points. The observed peak value is likely to be less that the true peak value and could occur before the true peak, in which case the slope could be affected. Blue line indicates the projected waning after the observed peak value. Ln, natural log; NC, nucleocapsid; S/CO, signal-to-cutoff value.

For this study, we analyzed S/CO values from the nucleocapsid antibody test starting from the day of the highest observed value (i.e., peak) (Figure 1). We converted all nucleocapsid antibody S/CO values to the natural log scale to capture the exponential decay as a linear function of the percentage change in S/CO per day. We classified donors as seroreverting if any donation crossed into nonreactivity (S/CO <1) at any point after their observed peak S/CO date.

To evaluate the consistency of the slope of decrease of S/CO values and to demonstrate the relationship of observed peak value to negative signal, we placed donors into deciles based on the natural log of their observed peak S/CO. We calculated days to seroreversion as the natural log of the peak S/CO divided by the estimated slope.

### Statistical Considerations

We used linear mixed effects regression to estimate the slope of nucleocapsid S/CO values from the observed peak. Demographic covariates serving as fixed effects were sex, age, and BMI at observed peak S/CO and binary race variable (Caucasian vs. non-Caucasian). We considered the intercept and days after peak S/CO as random effects to account for variations in observed peak S/CO values and follow-up time between donations, respectively. We used model influence diagnostics (restricted likelihood distance) to identify data points with large deviations from predicted values. We matched donors to those data points and their historical serology. We removed large increases in antibody nucleocapsid S/CO after an observed pattern of decrease from analysis, along with subsequent observations, because they were assumed to indicate reinfection.

We compared demographics of seroreverting donors at the time of peak donation (with BMI calculated from height and weight measurements) to nonseroreverting donors by using the G-test of independence for categorical variables and t-tests for continuous variables. We manually selected variables by using a forward stepwise process where each variable was first entered into a model with only days postpeak, after which significant variables were added one-by-one to build the final model. Vaccination status at peak S/CO was initially significant but was no longer significant after addition of the age, male, and Caucasian variables.

We evaluated the validity of the linear model by comparing model outcomes with actual observations among 3 groups of 10 randomly selected donors from different quartiles of observed peak values. We used SAS version 9.4 (SAS Institute Inc., https://www.sas.com) for all analyses.

## Results

### Study Population

A total of 51,414 donors (190,983 donations) seroconverted to nucleocapsid antibody reactivity during the universal screening period, of which 48,330 donations from 19,323 donors remained eligible for analysis (Tables 1, 2). The population had slightly more female than male donors (51.6% vs. 48.4%), a mean age of 52.3 years (SD 13.9 years), and was primarily of Caucasian race (94.6%); most (53.2%) persons resided in the Midwest United States and were overweight (mean BMI 28.8 kg/m^2^, SD 5.75 kg/m^2^). A significant difference was found in mean follow-up time between male donors (99.9 days, SD 48.05 days) and female donors (103.3 days, SD 48.08 days) (p<0.0001) although such a small difference is unlikely to have any effect in the model. A total of 145 (0.75%) persons seroreverted during the study. Persons who were older and had higher BMIs had higher S/CO values (albeit minor) when seroreverters and persons who did not serorevert were compared (Table 2). The effect of vaccination on seroreversion was not apparent in the model.

**Table 2:** Study sample donor demographics stratified by seroreversion status during the follow-up period for study of trajectory and demographic correlates of antibodies to SARS-CoV-2 nucleocapsid in recently infected blood donors, United States, June 2020‒June 2021*

Characteristic	Total, n = 19,323	Nonseroreverter, n = 19,178	Seroreverter,† n = 145	Significance‡
Sex				NS
F	9,965 (51.6)	9,890 (51.6)	75 (51.7)	
M	9,358 (48.4)	9,288 (48.4)	70 (48.3)	
Race/ethnicity				NS
Caucasian	18,285 (94.6)	18,143 (94.6)	142 (97.9)	
African American	221 (1.1)	220 (1.2)	1 (0.7)	
Hispanic	429 (2.2)	428 (2.2)	1 (0.7)	
American Indian	46 (0.2)	46 (0.2)	0	
>1 race	101 (0.5)	101 (0.5)	0	
Other	53 (0.3)	53 (0.3)	0	
Asian	159 (0.8)	159 (0.8)	0	
Missing	29 (0.2)	28 (0.2)	1 (0.7)	
Region				NS
Northeast	2,228 (11.5)	2,215 (11.6)	13 (8.9)	
Midwest	10,274 (53.2)	10,199 (53.2)	75 (51.7)	
South	3,998 (20.7)	3,965 (20.7)	33 (22.8)	
West	2,818 (14.6)	2,794 (14.6)	24 (16.6)	
Missing	5 (0.03)	5 (0.03)	0	
Mean age, y (SD)	52.3 (13.9)	52.3 (13.9)	49.6 (13.6)	p = 0.01
BMI, kg/m^2^ (SD)	28.8 (5.7)	28.8 (5.7)	27.4 (5.4)	p<0.01

*Values are no. (%) unless otherwise indicated. BMI, body mass index; NS, not significant; S/CO, signal-to-cutoff value. †Donor with any donation after the peak S/CO with loss of nucleocapsid reactivity (S/CO <1.0). ‡G-test of independence was used for categorical significance tests comparing demographics of seroreverting and nonseroreverting donors.

### Observed Peak S/CO and Rate of Waning

**Figure 2:**
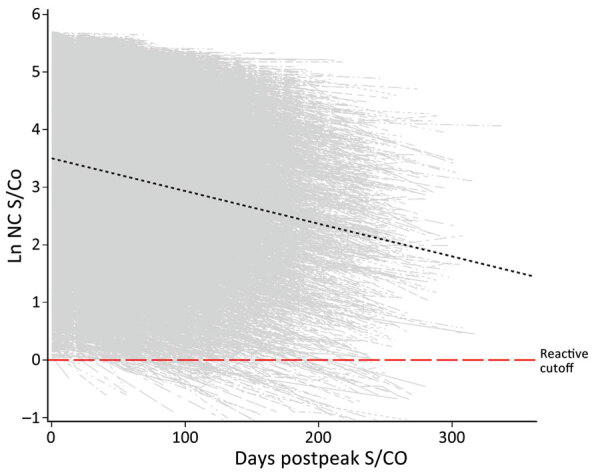
Longitudinal plot of all nucleocapsid-reactive donors with predicted slope (black dashed line) at mean observed peak S/CO in study of trajectory and demographic correlates of antibodies to SARS-CoV-2 nucleocapsid in recently infected blood donors, United States, June 2020‒June 2021. Shown are the overall dataset and a spaghetti plot of regression lines of S/CO values for each donor in the cohort. The natural log of observed peak values covered a range from ≈0 to ≈5.7, and the mean observation time was 101.7 days. Ln, natural log; NC, nucleocapsid; S/CO, signal-to-cutoff value.

The observed peak S/CO varied greatly between donors; on the natural log scale, the overall mean peak S/CO was 3.8 (SD 1.19), translating to a raw value of 44.7 (Figure 2), which also showed a relatively constant rate of waning across all donors. The mean observation period postpeak for all donors was 101.7 days. Using our linear model, we estimated peak values and identified differences by demographics in estimated peak S/CO values. For every additional year of age, the estimated peak S/CO increased by 1.4%. Caucasian persons were estimated to have a 28.6% lower peak S/CO than persons of other races. Every point increase in BMI was associated with an increased estimated peak S/CO by 3.4%. Male donors were estimated to have a 29.0% higher peak S/CO than female donors.

Linear mixed effects regression (Table 3) adjusting for age, sex, race, and BMI at peak S/CO estimated the population mean nucleocapsid antibody slope of decrease to be −0.656% (95% CI −0.64% to −0.67%) per day (p<0.0001). An interaction between age and days postpeak S/CO indicates the estimated S/CO decrease is slower as age increases, reducing the slope by 0.0017% (95% CI 0.0013%‒0.0021%) with each additional year of age. Donors were placed into deciles (Table 4) based on the natural log of their observed peak S/CO with days to seroreversion calculated as the natural log of the peak S/CO divided by the estimated slope using the sum of the population mean slope (−0.00656) and the age interaction slope using the mean age (0.000017 × 52), resulting in a slope of −0.568% per day, or a 122-day half-life.

**Table 3:** Parameter estimates modeling repeat donor nucleocapsid S/CO for study of trajectory and demographic correlates of antibodies to SARS-CoV-2 nucleocapsid in recently infected blood donors, United States, June 2020‒June 2021*

Parameter	Estimate (SE)	p value
Intercept	2.3278 (0.06794)	<0.0001
Days postpeak S/CO	−0.00656 (0.00009)	<0.0001
Male sex	0.2901 (0.08703)	0.0009
Caucasian race	−0.2856 (0.02960)	<0.0001
Age at peak S/CO	0.01445 (0.00061)	<0.0001
BMI at peak S/CO	0.03441 (0.00193)	<0.0001
Male† BMI at peak S/CO	−0.00834 (0.00295)	0.0047
Days postpeak† age at peak S/CO	0.000017 (0.000002)	<0.0001

*BMI, body mass index; S/CO, signal-to-cutoff value. †Multiply estimates by 100 to obtain percentage change per unit increase. Only effects that include days postpeak S/CO affect the slope of decrease where others effect peak S/CO.

**Table 4:** Days to seroreversion by peak nucleocapsid S/CO at a mean age of 52 for study of trajectory and demographic correlates of antibodies to SARS-CoV-2 nucleocapsid in recently infected blood donors, United States, June 2020‒June 2021*

Percent decile	Peak S/CO Ln (raw)	Days to 100% seroreversion
100	5.71 (301.87)	1,005
90	5.19 (179.47)	914
80	4.91 (135.64)	865
70	4.64 (103.54)	817
60	4.34 (76.71)	765
50	4.03 (56.26)	709
40	3.70 (40.45)	652
30	3.29 (26.84)	580
20	2.80 (16.44)	494
10	2.11 (8.25)	372

*Ln, natural log; S/CO, signal-to-cutoff value.

**Figure 3:**
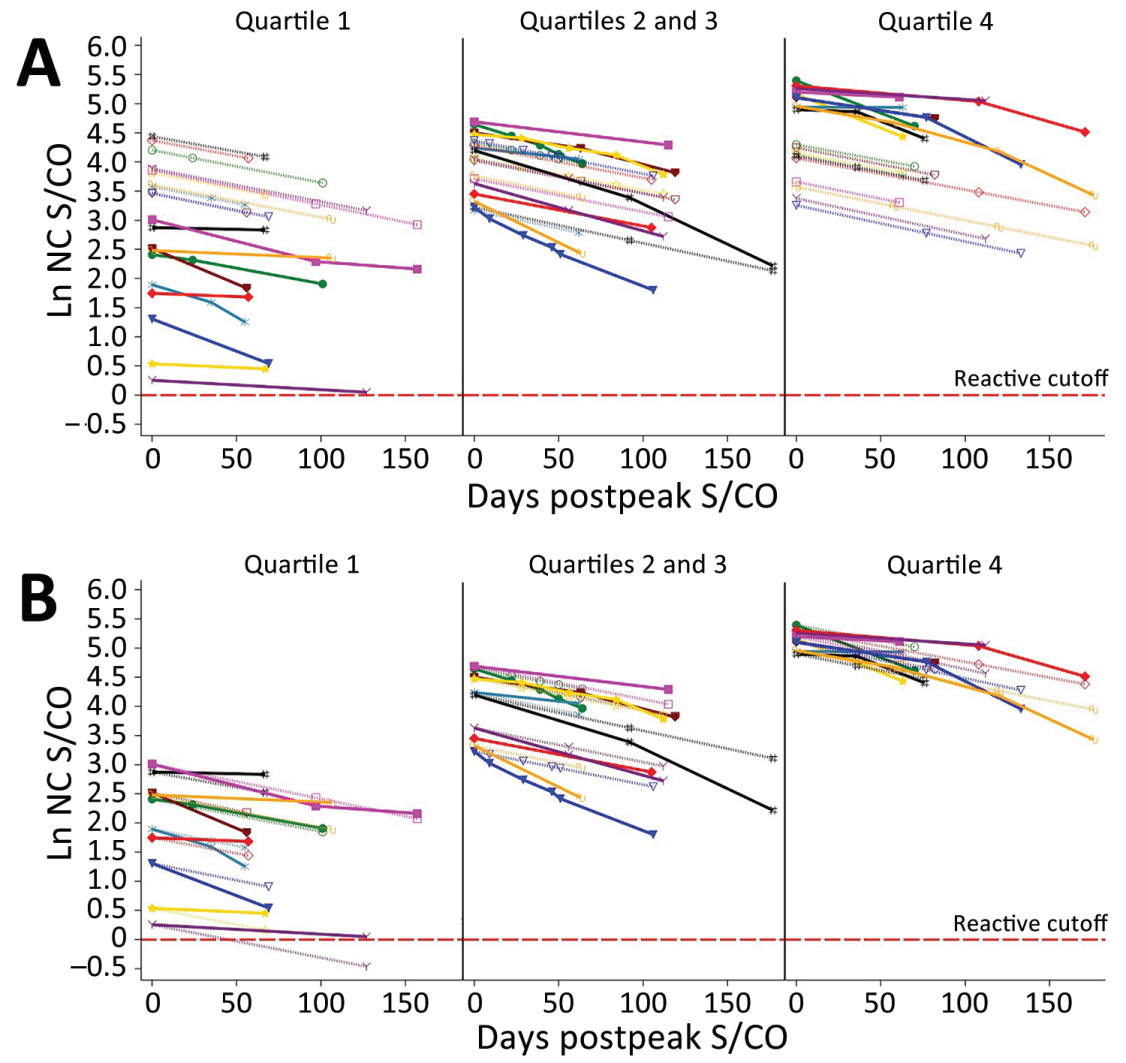
Predicted values and actual S/CO values. A) Raw predicted peak S/CO values and actual donor serologic values by peak S/CO quartile (n = 30); B) intercept-adjusted predicted S/CO values and actual donor serologic values by peak S/CO quartile (n = 30). Dotted lines indicate predicted values and solid lines observed values. Quartiles were defined on the basis of their observed peak value. Samples were randomly chosen. Ln, natural log; NC, nucleocapsid; S/CO, sample-to-cutoff value.

On the basis of the estimated decrease, donors are projected to remain reactive between 1 year and 2.75 years postpeak. To evaluate the efficacy of the linear model, we assigned donors to quartile groups based on peak S/CO. Ten donors from each quartile group were randomly selected, and their data were plotted with their observed and predicted serology values. We compiled raw model estimates of peak S/CO values (Figure 3, panel A) and adjustments of each of the estimated values by the difference in the observed and predicted peak S/CO to better depict the accuracy of the projected slope (Figure 3, panel B).

### Seroreversion

**Figure 4:**
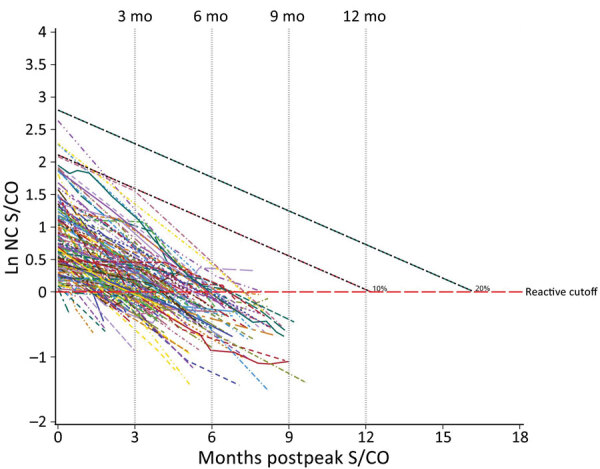
Longitudinal plot of nucleocapsid reactive seroreverting donors in study of trajectory and demographic correlates of antibodies to SARS-CoV-2 nucleocapsid in recently infected blood donors, United States, June 2020‒June 2021. All seroreverting donors are plotted by their observed testing values. Black dashed lines are overlaid as indicators of the first and second deciles of peak S/COs across all donors to show low starting values of those who seroreverted. Decile lines are plotted by using the mean age (52 years) of donors, but seroreverting donors were slightly younger (49 years) and showed faster waning. Ln, natural log; NC, nucleocapsid; S/CO, signal-to-cutoff value.

Nucleocapsid antibody serology for all seroreverters (n = 145, 0.75%) during the study period (Figure 4) showed 98% had peak S/CO values within the first decile (raw nucleocapsid antibody S/CO<8.25). The remaining values fell within the second decile (raw nucleocapsid antibody S/CO <16.44). We also determined the estimated time of waning to a negative test signal for 100% of donors in each decile (Table 4).

## Discussion

This large study supports the finding that, using the VITROS Anti-SARS-CoV-2 total nucleocapsid antibody test, the signal decreases with time, but the degree of decrease varies with donor age, as indicated by the age and days interaction. All other variables in the model contribute solely to the estimated peak. Therefore, using a linear model, we estimated the rate of decrease to be 0.656%/day, or a half-life of ≈106 days, or 122 days if correction is made for the age interaction (i.e., as age increases, waning decreases), using the average donor age in the study. Direct observation of the study population showed that only 0.75% of study participants seroreverted, all of whom had an observed peak value within the 2 lowest deciles for the population. The validation study of the linear model did not accurately define the peak signal level (Figure 3, panel A), indicating that peak signal levels are inherently variable over a wide range. However, when the model was adjusted to compare the waning slopes, these slopes were found to be consistent across a range of different observed peak values (Figure 3, panel B).

Our results are similar to those from a longitudinal study of employees at Brigham and Women’s Hospital (Boston, MA, USA), where it was estimated that the nucleocapsid antibody half-life was 128 days (*8*), compared to our study value of 122 days. We also observed a similar interaction effect between nucleocapsid antibody levels and BMI in men as that reported by Yamamoto et al. (*10*). Our study demonstrated that the Roche Elecsys Total Ig nucleocapsid test for serosurveillance of SARS-CoV-2 as antibody reactivity remains detectable for >1 year in 90% and >2 years in 50% of donors in our cohort. Our results contrast those of Alfego et al., who found a decrease of 31.8% nucleocapsid seropositivity by using a qualitative assay after 293 days in a population-based analysis (*11*). The explanation for this difference is probably low peak antibody levels in that study’s population.

Although immunity to SARS-CoV-2 wanes over time, the extent to which this waning is linked to nucleocapsid antibody signal strength is unknown and should not be assumed. However, the trajectory of antibody levels is such that an estimate of seroreversion may be made for any given population if the distribution of observed peak values is known. In addition, the same information could be used to estimate the number of persons who seroconverted at a given time in the past.

Strengths of our study include a large geographically dispersed sample within the United States, with almost equal representation by sex, and the ability to isolate incident infections on the basis of previous nonreactive results. Limitations of the study include that blood donors are mostly Caucasian, are healthy at the time of donation, and are not fully representative of the general US population. In addition, during the study period, a higher proportion of blood donors were vaccinated relative to the population at large (*2*). Nevertheless, donors have been used in this context for previous research (*2*). A second limitation is that although a linear model appears to be appropriate, there was some deviation from linearity among donors who had the highest and lowest antibody S/CO signal levels. For donors who had low levels, this finding may reflect the inherent variability of signal levels close to the cutoff value of the test used. Geography, grouped by US census region, was not considered for the model because it was believed to only affect the timing of infection onset, as opposed to providing a biologic effect on S/CO values. In addition, the method did not necessarily identify true peak signal levels. Nevertheless, it is clear that the range of signal levels was highly variable, but the slope of decrease was similar across all observed peak levels.

In conclusion, we evaluated the waning of signal levels for SARS-CoV-2 nucleocapsid antibodies in a large cohort of blood donors by using a widely available total antibody test. Waning rates were consistent across a wide range of observed signal peaks. Rates differed somewhat by age and BMI. The overall half-life of the antibody signal was 106 days uncorrected and 122 days when corrected for age and BMI distribution in the study cohort. We believe that the method reported here may be generalized to other antibody systems.
